# Impact of baseline COPD symptom severity on the benefit from dual *versus* mono-bronchodilators: an analysis of the EMAX randomised controlled trial

**DOI:** 10.1177/1753466620968500

**Published:** 2020-11-09

**Authors:** Claus F. Vogelmeier, Edward M. Kerwin, Leif H. Bjermer, Lee Tombs, Paul W. Jones, Isabelle H. Boucot, Ian P. Naya, David A. Lipson, Chris Compton, Neil Barnes, François Maltais

**Affiliations:** Department of Medicine, Pulmonary and Critical Care Medicine, University Medical Centre Giessen and Marburg, Philipps-Universität Marburg, Germany; Member of the German Centre for Lung Research (DZL), Baldingerstraße, Marburg 35043, Germany; Clinical Research Institute of Southern Oregon, Medford, OR, USA; Respiratory Medicine and Allergology, Lund University, Lund, Sweden; Precise Approach Ltd, Contingent Worker on Assignment at GSK, Stockley Park West, Uxbridge, Middlesex, UK; GSK, Brentford, Middlesex, UK; GSK, Brentford, Middlesex, UK; GSK, Brentford, Middlesex, UK; RAMAX Ltd., Bramhall, Cheshire, UK; Respiratory Clinical Sciences, GSK, Collegeville, PA, USA; Perelman School of Medicine, University of Pennsylvania, Philadelphia, PA, USA; GSK, Brentford, Middlesex, UK; GSK, Brentford, Middlesex, UK; Centre de Pneumologie, Institut universitaire de cardiologie et de pneumologie de Québec, Université Laval, Québec, Québec, Canada

**Keywords:** bronchodilator therapy, CAT, COPD, symptoms, umeclidinium/vilanterol

## Abstract

**Rationale:**

Symptom relief is a key treatment goal in patients with chronic obstructive pulmonary disease (COPD). However, there are limited data available on the response to bronchodilator therapy in patients at low risk of exacerbations with different levels of symptom severity. This study compared treatment responses in patients with a range of symptom severities as indicated by baseline COPD assessment test (CAT) scores.

**Methods:**

The 24-week EMAX trial evaluated the benefits of umeclidinium/vilanterol *versus* umeclidinium or salmeterol in symptomatic patients at low exacerbation risk who were not receiving inhaled corticosteroids. This analysis assessed lung function, symptoms, health status, and short-term deterioration outcomes in subgroups defined by a baseline CAT score [<20 (*post hoc*) and ⩾20 (pre-specified)]. Outcomes were also assessed using *post hoc* fractional polynomial modelling with continuous transformations of baseline CAT score covariates.

**Results:**

Of the intent-to-treat population (*n* = 2425), 56% and 44% had baseline CAT scores of <20 and ⩾20, respectively. Umeclidinium/vilanterol demonstrated favourable improvements compared with umeclidinium and salmeterol for the majority of outcomes irrespective of the baseline CAT score, with the greatest improvements generally observed in patients with CAT scores <20. Fractional polynomial analyses revealed consistent improvements in lung function, symptoms and reduction in rescue medication use with umeclidinium/vilanterol *versus* umeclidinium and salmeterol across a range of CAT scores, with the largest benefits seen in patients with CAT scores of approximately 10–21.

**Conclusions:**

Patients with symptomatic COPD benefit similarly from dual bronchodilator treatment with umeclidinium/vilanterol. Fractional polynomial analyses demonstrated the greatest treatment differences favouring dual therapy in patients with a CAT score <20, although benefits were seen up to scores of 30. This suggests that dual bronchodilation may be considered as initial therapy for patients across a broad range of symptom severities, not only those with severe symptoms (CAT ⩾20).

**Trial registration:** NCT03034915, 2016-002513-22 (EudraCT number).

*The reviews of this paper are available via the supplemental material section.*

## Introduction

Inhaled bronchodilator treatment is the foundation of maintenance therapy for chronic obstructive pulmonary disease (COPD). Dual bronchodilator therapy with long-acting muscarinic agonist/long-acting β_2_-agonist (LAMA/LABA) combinations provides greater improvements in lung function than LAMA or LABA monotherapy, although the extent of improvements in symptoms and health status varies between studies.^1–8^ Dual bronchodilation can also reduce the risk of a short-term deterioration, as demonstrated using the clinically important deterioration (CID) composite endpoint, compared with inhaled corticosteroid (ICS)/LABA combinations or bronchodilator monotherapy.^2,8–12^

The Global Initiative for Chronic Obstructive Lung Disease (GOLD) 2020 strategy report bases treatment recommendations on the assessment of symptoms using the modified Medical Research Council (mMRC) questionnaire and the COPD Assessment Test (CAT), and exacerbation risk.^13^ Patients with CAT scores ⩾10 show a significant impact of their symptoms on their health, wellbeing and daily life.^14^ A CAT score ⩾10 or mMRC score ⩾2 have been suggested as the threshold for considering maintenance treatment for COPD;^13^ however, there is evidence that these thresholds do not necessarily correspond to similar levels of disease burden.^15^

Dual bronchodilator therapy is recommended by the GOLD 2020 strategy report as initial maintenance treatment for symptomatic patients at low risk of exacerbations (GOLD B) who have severe breathlessness, or for symptomatic patients (CAT scores ⩾20) at high risk of exacerbations (GOLD D).^13^ These recommendations are based on a pooled *post hoc* analysis of two clinical trials showing numerically greater relative benefits of LAMA/LABA treatment on the St George’s Respiratory Questionnaire (SGRQ) score compared with placebo in patients with higher CAT scores.^16^ However, when compared with bronchodilator monotherapy, the same analysis showed similar incremental benefits with LAMA/LABA across the full range of CAT scores.^16^ In addition, neither of the two trials included in the analysis prospectively demonstrated a symptomatic benefit of dual bronchodilation on daily total symptom score compared with bronchodilator monotherapy.^17^ Consequently, there remains a need for further evidence of the incremental benefits associated with bronchodilator therapies in patients with COPD across varying levels of symptom severity.

The Early MAXimisation of bronchodilation for improving COPD stability (EMAX) trial examined the benefits of dual bronchodilation with umeclidinium/vilanterol (UMEC/VI) *versus* UMEC or salmeterol (SAL) monotherapy in symptomatic patients at low exacerbation risk who were not receiving ICS.^8^ UMEC/VI demonstrated consistent statistically significant improvements over UMEC and SAL in lung function and symptomatic outcomes as well as a significant reduction in the risk of CID compared with either monotherapy.^8^ This subgroup analysis of the EMAX trial compared UMEC/VI with both monotherapies on improvements in lung function, patient-reported symptom severity and health status, and short-term disease deterioration (as measured by CID) outcomes according to the baseline CAT score to determine whether symptom severity at baseline may be associated with treatment responses to dual *versus* mono-bronchodilator therapy.

## Materials and methods

### Study design and patients

In the multicentre, randomised, double-blind, double-dummy, three-arm parallel group EMAX trial (NCT03034915; GSK study number 201749), patients were randomly allocated 1:1:1 to once-daily UMEC/VI 62.5/25 µg *versus* the ELLIPTA inhaler, once-daily UMEC 62.5 µg *versus* ELLIPTA, or twice-daily SAL 50 µg *versus* the DISKUS inhaler.^8^ Full methodology has been published previously.^8^ Eligible patients were ⩾40 years of age and were current/former smokers (⩾10 pack-years smoking history) with a COPD diagnosis, pre and post-salbutamol forced expiratory volume in 1 second/forced vital capacity (FEV_1_/FVC) ratio <0.7, post-salbutamol FEV_1_ of ⩾30 to ⩽80% predicted, CAT score ⩾10, and ⩽1 moderate exacerbation and no severe exacerbations in the previous year. Before screening and during the 4-week run-in period, bronchodilator maintenance therapy was limited to a LAMA or LABA only, with no other COPD maintenance medications permitted. All patients were free of ICS and ICS/LABA for ⩾6 weeks and free of LAMA/LABA for ⩾2 weeks prior to run-in. As needed salbutamol was permitted throughout the study.

The trial was conducted according to the principles of the Declaration of Helsinki and received appropriate ethical approval. All patients provided written informed consent. This manuscript conforms to the CONSORT guidelines for publication of randomised controlled trials (Supplemental Table 1).

### Endpoints and assessments

The primary endpoint for this study was change from baseline in trough FEV_1_ at week 24. Patient-reported symptom-based assessments included the self-administered computerised transition dyspnoea index (SAC-TDI), evaluating respiratory symptoms-COPD (E-RS) total score, daily rescue salbutamol use, global assessment of disease severity (GADS), and CAT score. Health status was assessed using the SGRQ total score. GADS was captured using a 7-point Likert scale ranging from ‘much better’ to ‘much worse’ compared with baseline. Responders were prospectively defined as individual patients with ⩾1-point improvement in SAC-TDI score,^18^ ⩾2-point reduction from baseline in E-RS total score,^19^ ⩾4-point reduction from baseline in SGRQ total score,^20^ and ⩾2-unit improvement from baseline in CAT score.^21^ A *post hoc* responder analysis evaluated the proportion of patients with a ⩾100 mL increase in trough FEV_1_ from baseline.

The risk of a first CID was assessed in individual patients according to three composite definitions: (A) a first moderate or severe exacerbation, and/or a decrease in trough FEV_1_ ⩾100 mL, and/or a deterioration in health status indicated by a decrease in SGRQ score of ⩾4 units from baseline; (B) the same as definition A, but with a decrease in CAT score of ⩾2 units from baseline replacing the SGRQ deterioration; (C) an FEV_1_-free definition including a first moderate or severe exacerbation, and/or a SGRQ deterioration, and/or a CAT deterioration, and/or a deterioration in SAC-TDI score of ⩾1 unit from baseline. Moderate exacerbations were defined as those necessitating treatment with oral corticosteroids and/or antibiotics; severe exacerbations were defined as those requiring hospitalisation or an emergency room visit.

### Statistical analysis

Results are presented for two baseline CAT score severity subgroups [<20 (*post hoc*) *versus* ⩾20 (pre-specified)].

Comparisons of baseline characteristics by baseline CAT score were analysed *post hoc* using *t*-tests for continuous variables and Fisher’s exact test for proportions of categorical variables. For the primary endpoint, change from baseline in trough FEV_1_ at week 24 was analysed using a mixed model repeated measures (MMRM) analysis with covariates of baseline FEV_1_, geographical region, number of bronchodilators during run-in (none or 1), visit, treatment, and visit by baseline and visit by treatment interactions (where visit is nominal). Least squares (LS) mean and LS mean change from baseline with estimated treatment differences and 95% confidence intervals (CIs) are reported.

Responder analysis was performed using a generalised linear mixed model with treatment as an explanatory variable and covariates of visit, baseline FEV_1_, number of bronchodilators during run-in (none or 1), geographical region, and visit by baseline score and visit by treatment interactions (where visit is nominal). Corresponding odds ratios (ORs) are reported with 95% CIs. Hazard ratios (HRs) and 95% CIs for the time to first CID were based on a Cox proportional hazards model with covariates of treatment, stratum (number of bronchodilators per day during run-in), geographical region, trough FEV_1_ at baseline, and SGRQ score at baseline.

A complementary *post hoc* analysis used a fractional polynomial model with CAT score as a continuous variable. These analyses were conducted across baseline CAT scores of 10–30 due to the distribution of CAT scores within the study population, which included few patients with scores outside this range. The best fitting fractional polynomial (FP) model from the FP(2) class is presented. The fitted MMRM included covariates of baseline FEV_1_, geographical region, number of bronchodilators per day during run-in, visit, treatment, FP1, FP2, and visit by baseline FEV_1_, visit by treatment, FP1*treatment and FP2*treatment interactions. FP1 and FP2 represent continuous power transformations of CAT score at baseline.

Unless stated otherwise, other efficacy endpoints were analysed in a similar manner; for analyses of the E-RS score and rescue medication use, the 4-weekly period was included as a covariate in place of visit.

## Results

### Patient disposition and demographics

Of the 2425 patients in the intent-to-treat (ITT) population (UMEC/VI: *n* = 812; UMEC: *n* = 804; SAL: *n* = 809), 56% and 44% of patients had baseline CAT scores of <20 and ⩾20, respectively, with similar proportions across treatment arms. There were significant differences in the baseline characteristics of the CAT <20 and CAT ⩾20 subgroups; for example, 24% and 39% of patients did not receive maintenance treatment during run-in, mean E-RS total scores were 8.4 and 13.4 and mean baseline SGRQ scores were 36.8 and 54.7, respectively (Table 1). In addition, 593 (44%) and 610 (57%) patients were current smokers in the CAT <20 and CAT ⩾20 subgroups at baseline, respectively. Overall, 317 (23%) and 308 (29%) patients were reversible to salbutamol in the CAT <20 and CAT ⩾20 subgroups, respectively. Mean rescue medication use was 1.6 and 2.8 puffs/day in the CAT <20 and CAT ⩾20 subgroups, respectively. During the run-in period, 1576/2425 (65%) of patients in the ITT population were receiving long-acting bronchodilator maintenance medication [LAMA: 1194/2425 (49%); LABA: 404/2425 (17%)].

**Table 1.** Patient demographics and baseline characteristics.

Characteristic	ITT (*n* = 2425)	CAT <20 (*n* = 1352)	CAT ⩾20 (*n* = 1073)	*p*-value CAT <20 *versus* ⩾20
Age, years, mean (SD)	64.6 (8.5)	65.5 (8.2)	63.5 (8.7)	<0.001
Female, *n* (%)	988 (41)	513 (38)	475 (44)	0.002
No maintenance treatment during run-in, n (%)	749 (31)	329 (24)	420 (39)	<0.001
Moderate COPD exacerbation history in prior year,^a^ *n* (%)	393 (16)	243 (18)	150 (14)	0.009
Duration of COPD, years, mean (SD)	8.3 (6.6)	7.9 (6.3)	8.8 (6.8)	<0.001
Post-salbutamol % predicted FEV_1_, mean (SD)	55.4 (12.7)	56.1 (12.5)	54.6 (13.0)	0.005
Baseline CAT score, *n* (%)				
<20	1352 (56)	1352 (100)	0	–
⩾20	1073 (44)	0	1073 (100)	–
Baseline CAT score, mean (SD)	19.2 (6.1)	14.7 (2.8)	24.9 (4.1)	–
BDI score, mean (SD)	7.0 (1.9)	7.6 (1.7)	6.3 (1.8)	<0.001
Baseline E-RS total score	10.6 (5.7)	8.4 (4.7)	13.4 (5.7)	<0.001
Baseline SGRQ score, mean (SD)	44.7 (16.2)	36.8 (12.4)	54.7 (14.8)	<0.001

a Number of exacerbations requiring oral or systemic corticosteroids and/or antibiotics (moderate) in 12 months prior to screening [patients with >1 moderate exacerbation or with a severe exacerbation (requiring hospitalization) were excluded].

BDI, baseline dyspnoea index; CAT, COPD assessment test; COPD, chronic obstructive pulmonary disease; E-RS, evaluating respiratory symptoms COPD; FEV_1_, forced expiratory volume in 1 second; FVC, forced vital capacity; ITT, intent-to-treat; SD, standard deviation; SGRQ, St George’s respiratory questionnaire.

### CAT subgroup analyses

#### Trough FEV_1_

**Figure 1.**
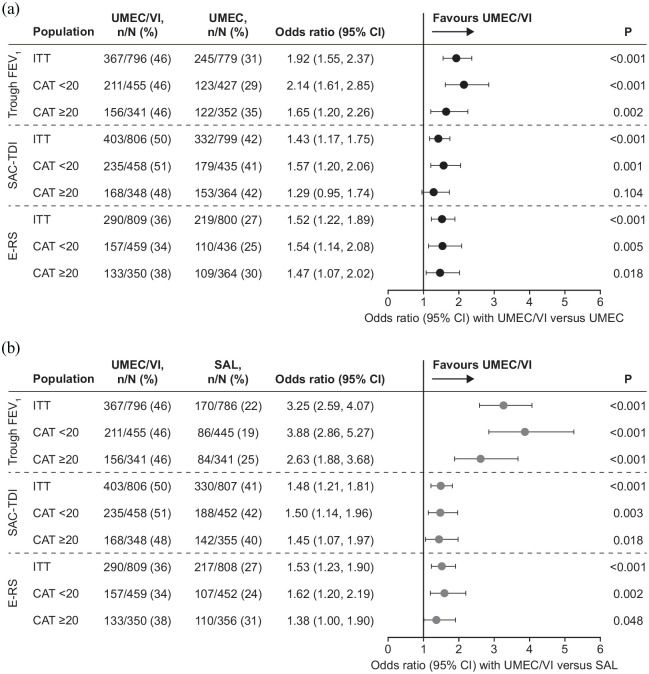
Proportion of responders for trough FEV_1_,^a^ SAC-TDI,^a^ and E-RS^b^ with UMEC/VI *versus* (a) UMEC and (b) SAL. ^a^Analyses conducted using a generalised linear model with treatment as an explanatory variable and covariates of visit, baseline trough FEV_1_/SAC-BDI focal score, number of bronchodilators received during run-in (0 or 1), geographical region, and visit by baseline FEV_1_/SAC-BDI focal score and visit by treatment interactions. ^b^Analyses conducted using a generalised linear model with treatment as an explanatory variable and 4-weekly period, baseline E-RS score, number of bronchodilators received during run-in (0 or 1), geographical region, and visit by baseline E-RS score and visit by treatment interactions. CAT, COPD assessment test; CI, confidence interval; E-RS, evaluating respiratory symptoms-COPD; FEV_1_, forced expiratory volume in 1 second; ITT, intent-to-treat; *n*/*N*, number of responders/number of patients with analysable data; SAC-TDI, self-administered computerised transition dyspnoea index; SAL, salmeterol; UMEC, umeclidinium; VI, vilanterol.

For both CAT subgroups, UMEC/VI demonstrated greater improvements from baseline in trough FEV_1_ at week 24 than monotherapy; statistically significant treatment differences favoured UMEC/VI (*versus* UMEC: 64–67 mL; *versus* SAL: 110–164 mL; all *p* < 0.001) and were numerically similar to those observed in the ITT population (Table 2). For both subgroups, the proportions of responders for FEV_1_ favoured UMEC/VI over monotherapy with ORs of 1.65–3.88 (*p* < 0.01), which were similar to those in the ITT population, with numerically higher ORs in the CAT <20 subgroup *versus* the CAT ⩾20 subgroup (Figure 1).

**Table 2.** LS mean change from baseline by subgroup for lung function and symptoms with UMEC/VI *versus* UMEC and SAL.

	ITT population			CAT <20 subgroup			CAT ⩾20 subgroup		
	UMEC/VI (*n* = 812)	UMEC (*n* = 804)	SAL (*n* = 809)	UMEC/VI (*n* = 461)	UMEC (*n* = 438)	SAL (*n* = 453)	UMEC/VI (*n* = 351)	UMEC (*n* = 366)	SAL (*n* = 356)
*Trough FEV_1_ at week 24, mL* ^a^									
LS mean change from baseline (95% CI)	122 (106, 138)	56 (39, 73)	–19 (–35, –2)	118 (97, 139)	51 (29, 73)	–46 (–68, –25)	127 (102, 152)	63 (37, 88)	17 (–9, 42)
UMEC/VI *versus* comparator mean treatment difference (95% CI)	–	**66** **(43, 89), *p* < 0.001**	**141** **(118, 164)** ***p* < 0.001**	–	**67** **(37, 97)** ***p* < 0.001**	**164** **(135, 194)** ***p* < 0.001**	–	**64** **(29, 100)** ***p* < 0.001**	**110** **(75, 146)** ***p* < 0.001**
*SAC-TDI focal score at week 24* ^a^									
LS mean change (95% CI)	1.68 (1.46, 1.89)	1.30 (1.08, 1.53)	1.22 (1.00, 1.44)	1.78 (1.51, 2.04)	1.39 (1.11, 1.67)	1.19 (0.92, 1.46)	1.55 (1.20, 1.90)	1.19 (0.83, 1.54)	1.29 (0.94, 1.65)
UMEC/VI *versus* comparator mean treatment difference (95% CI)	–	**0.37 (0.06, 0.68) *p* = 0.018**	**0.45 (0.15, 0.76) *p* = 0.004**	–	0.39 (0.00, 0.77) *p* = 0.051	**0.58** **(0.20, 0.96)** ***p* = 0.003**	–	0.36 (–0.14, 0.87) *p* = 0.154	0.26 (–0.24, 0.76) *p* = 0.310
*4-Weekly E-RS total score at weeks 21–24* ^b^									
LS mean change from baseline (95% CI)	–1.52 (–1.81, –1.23)	–0.99 (–1.29, –0.69)	–0.69 (–0.98, –0.39)	–1.09 (–1.43, –0.75)	–0.65 (–1.00, –0.29)	–0.20 (–0.55, 0.14)	–2.04 (–2.55, –1.54)	–1.43 (–1.94, –0.92)	–1.30 (–1.81, –0.80)
UMEC/VI *versus* comparator mean treatment difference (95% CI)	–	**–0.53 (–0.95, –0.11) *p* = 0.013**	**–0.83 (–1.25, –0.42) *p* < 0.001**	–	–0.44 (–0.94, 0.05) *p* = 0.078	**–0.89** **(–1.37, –0.40)** ***p* < 0.001**	–	–0.61 (–1.33, 0.10) *p* = 0.092	**–0.74** **(–1.45, –0.02) *p* = 0.043**
*Rescue medication puffs/day over weeks 1–24* ^b^									
LS mean change from baseline (95% CI)	–0.61 (–0.71, –0.50)	–0.28 (–0.38, –0.17)	–0.32 (–0.43, –0.22)	–0.43 (–0.55, –0.31)	–0.03 (–0.15, 0.10)	–0.10 (–0.22, 0.02)	–0.82 (–0.99, –0.64)	–0.58 (–0.76, –0.41)	–0.61 (–0.78, –0.43)
UMEC/VI *versus* comparator mean treatment difference (95% CI)	–	**–0.33 (–0.48, –0.18) *p* < 0.001**	**–0.28 (–0.43, –0.14) *p* < 0.001**	–	**–0.41** **(–0.58, –0.23)** ***p* < 0.001**	**–0.33** **(–0.50, –0.16)** ***p* < 0.001**	–	–0.23 (–0.48, 0.02) *p* = 0.067	–0.21 (–0.46, 0.04) *p* = 0.102
*Global assessment of disease severity at week 24* ^c^									
Proportion of patients reporting improvement from baseline at week 24, *n*/*N* (%)	473/707 (67)	393/638 (62)	413/674 (61)	275/409 (67)	222/353 (63)	238/387 (62)	198/298 (66)	171/285 (60)	175/287 (61)
Ordered OR for better response category with UMEC/VI *versus* comparator (95% CI)	–	**1.38 (1.14, 1.67) *p* = 0.001**	**1.38 (1.14, 1.68) *p* < 0.001**	–	**1.42** **(1.10, 1.84)** ***p* = 0.008**	**1.41** **(1.09, 1.82)** ***p* = 0.008**	–	1.32 (0.99, 1.78) *p* = 0.061	1.32 (0.98, 1.77) *p* = 0.064

a Analyses conducted using MMRM with covariates of baseline FEV_1_/SAC-BDI focal score, geographical region, number of bronchodilators received during run-in (0 or 1), visit, treatment, and visit by baseline FEV_1_/SAC-BDI focal score and visit by treatment interactions (where visit is nominal).

b Analyses conducted using MMRM with covariates of baseline E-RS total score/baseline rescue medication use (puffs/day), geographical region, number of bronchodilators received during run-in (0 or 1), 4-weekly period, treatment, and 4-weekly period by baseline E-RS total score/baseline rescue medication use (puffs/day) and 4-weekly period by treatment interactions.

c Analysis performed separately at each visit using a generalised linear model with covariates of treatment, number of bronchodilators received during run-in (0 or 1), and geographical region.

BDI, baseline dyspnoea index; CAT, COPD assessment test; CI, confidence interval; COPD, chronic obstructive pulmonary disease; E-RS, evaluating respiratory symptoms-COPD; FEV_1_, forced expiratory volume in 1 second; ITT, intent-to-treat; LS, least squares; MMRM, mixed model repeated measures; OR, odds ratio; SAC, self-administered computerised; SGRQ, St George’s respiratory questionnaire; TDI, transition dyspnoea index; UMEC, umeclidinium; VI, vilanterol.

#### SAC-TDI and E-RS

In both subgroups, improvements from baseline were observed in the SAC-TDI focal score at week 24 and E-RS total score at weeks 21–24 for all treatment arms, with UMEC/VI demonstrating the greatest improvements (Table 2). Larger improvements from baseline in E-RS total score were observed in the highly symptomatic (CAT ⩾20) subgroup in all treatment groups. For both subgroups, treatment differences in SAC-TDI and E-RS were similar to the differences observed in the ITT population (Table 2).

The proportions of responders for SAC-TDI focal score favoured UMEC/VI *versus* UMEC in the CAT <20 subgroup (*p* = 0.001) and *versus* SAL in both subgroups (*p* < 0.05) (Figure 1). The proportions of responders for E-RS total score favoured UMEC/VI *versus* monotherapy in both subgroups (*p* < 0.05). For both SAC-TDI and E-RS, ORs for responders *versus* non-responders favoured UMEC/VI *versus* monotherapy (1.29−1.62) and were also similar to those in the ITT population (Figure 1).

#### Rescue medication use

The greatest reductions in rescue medication use from baseline over weeks 1−24 were seen with UMEC/VI compared with monotherapy in both subgroups (Table 2). In both subgroups, treatment differences favoured UMEC/VI, as seen in the ITT population, but were larger in the CAT <20 subgroup (−0.33 to −0.41; *p* < 0.001) than in the CAT ⩾20 subgroup (−0.21 to −0.23; not statistically significant [NS]) (Table 2).

#### GADS

A numerically greater proportion of patients receiving UMEC/VI rated their overall severity of COPD at week 24 as improved from baseline compared with monotherapy in both subgroups (Table 2). Treatment differences favoured UMEC/VI, as in the ITT population, and were larger in the CAT <20 subgroup (1.41−1.42; *p* = 0.008) than in the CAT ⩾20 subgroup (1.32; NS) (Table 2).

#### SGRQ and CAT

As in the ITT population, the proportions of SGRQ and CAT responders and ORs numerically favoured UMEC/VI *versus* monotherapy in both subgroups (Supplemental Figure 1). For SGRQ, as in the ITT population, greater increases in odds of responding were seen with UMEC/VI *versus* SAL in both subgroups (*p* < 0.05), but not *versus* UMEC. For CAT, greater increases in odds of responding with UMEC/VI *versus* UMEC or SAL were observed in the CAT <20 subgroup (34−42%; *p* < 0.05) compared with the CAT ⩾20 subgroup (11−27%; NS) (Supplemental Figure 1).

#### Short-term deterioration (CID)

**Figure 2.**
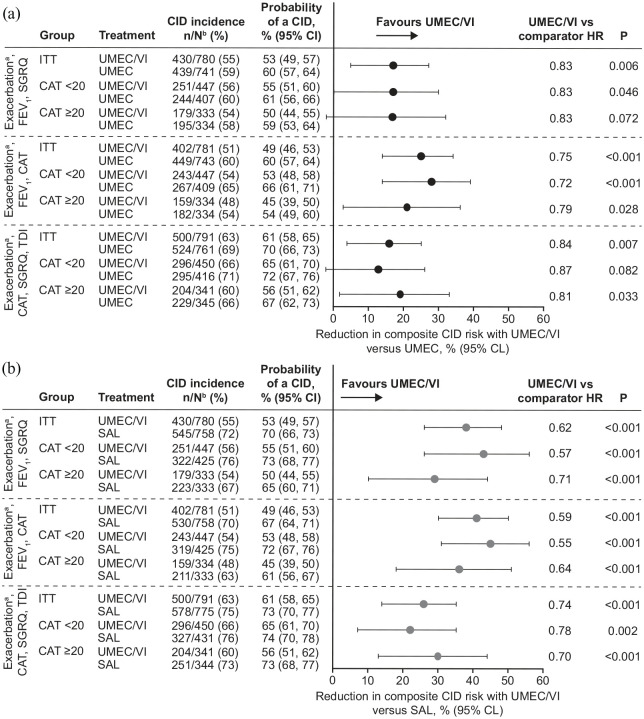
Risk of a first CID up to day 168 according to multiple composite definitions with UMEC/VI *versus* (a) UMEC and (b) SAL. Analyses conducted using a Cox proportional hazards model with covariates of treatment, number of bronchodilators received during run-in (0 or 1), geographical region, baseline trough FEV_1_ and baseline SGRQ score. ^a^Moderate/severe exacerbation. ^b^n/N, patients who experienced a CID/patients with ⩾1 post baseline assessment (not including exacerbations) for at least one of the individual components or patients who had an exacerbation. CAT, COPD assessment test; CI, confidence interval; CID, clinically important deterioration; FEV_1_, forced expiratory volume in 1 second; HR, hazard ratio; ITT, intent-to-treat; SGRQ, St George’s respiratory questionnaire; TDI, transition dyspnoea index; SAL, salmeterol; UMEC, umeclidinium; VI, vilanterol.

When considering all CID definitions, the incidence of CID was generally higher in the CAT <20 subgroup than in the CAT ⩾20 subgroup, particularly in the monotherapy treatment arms (Figure 2). The reductions in CID risk favoured UMEC/VI *versus* UMEC and SAL in both CAT subgroups according to all three definitions (13−45%), and were numerically similar to the ITT population (Figure 2). Larger reductions in risk were observed with UMEC/VI *versus* SAL in the ITT population and both subgroups (22−45%) than when compared with UMEC (13−28%).

### Fractional polynomial analyses with CAT as a continuous variable

#### Trough FEV_1_

**Figure 3.**
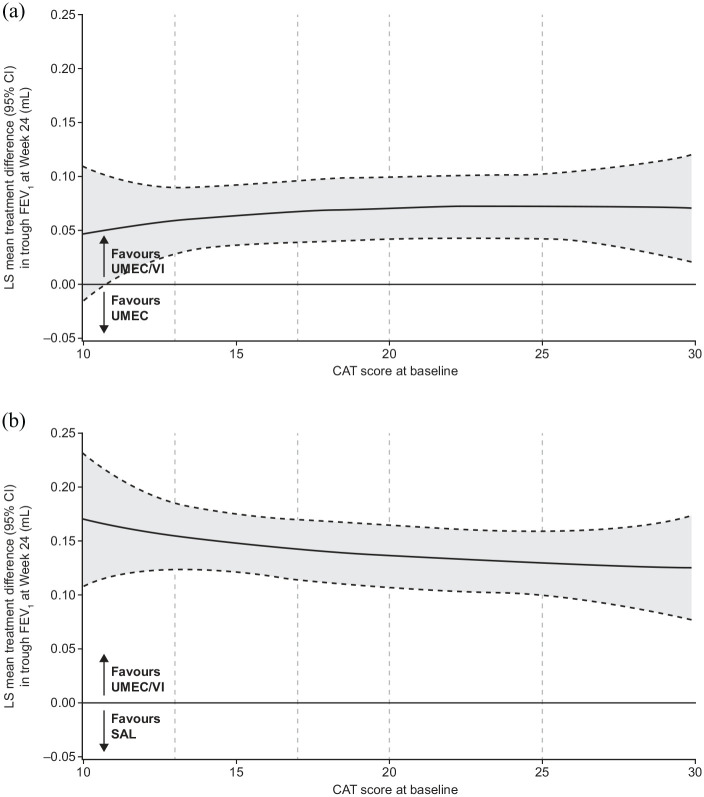
Improvement in trough FEV_1_ at week 24 by baseline CAT score with UMEC/VI *versus* (a) UMEC and (b) SAL. Vertical dotted lines indicate quintiles of CAT score at baseline. FP analyses were conducted across baseline CAT scores of 10–30 as the study population included few patients with scores outside this range. The fitted MMRM included covariates of baseline FEV_1_, geographical region, number of bronchodilators per day during run-in, visit, treatment, FP1, FP2, and visit by baseline FEV_1_, visit by treatment, FP1*treatment and FP2*treatment interactions. CAT, COPD assessment test; CI, confidence interval; FEV1, forced expiratory volume in 1 second; FP, fractional polynomial; LS, least squares; MMRM, mixed model repeated measures; SAL, salmeterol; UMEC, umeclidinium; VI, vilanterol.

In *post hoc* analyses, the lower bound of the 95% CI for improvements in trough FEV_1_ at week 24 excluded 0 for UMEC/VI and UMEC, but not for SAL (Supplemental Figure 2A). Consistent improvements in trough FEV_1_ were observed with UMEC/VI *versus* UMEC and SAL across the range of baseline CAT scores (Figure 3).

#### SAC-TDI and E-RS

**Figure 4.**
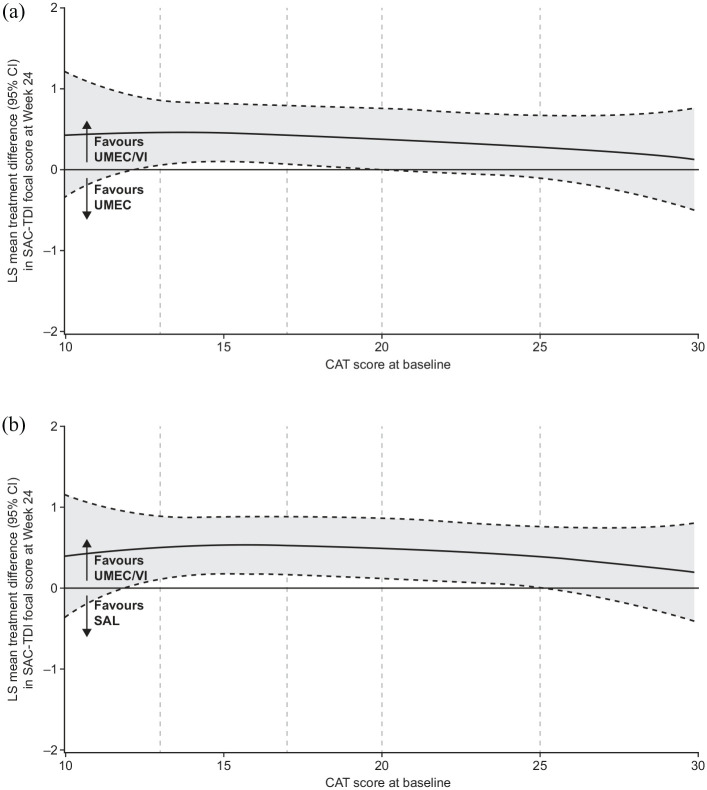
Improvement in SAC-TDI focal score at week 24 by baseline CAT score with UMEC/VI *versus* (a) UMEC and (b) SAL. Vertical dotted lines indicate quintiles of CAT score at baseline. FP analyses were conducted across baseline CAT scores of 10–30 as the study population included few patients with scores outside this range. The fitted MMRM included covariates of SAC-BDI, geographical region, number of bronchodilators per day during run-in, visit, treatment, FP1, FP2, and visit by SAC-BDI, visit by treatment, FP1*treatment and FP2*treatment interactions. BDI, baseline dyspnoea index; CAT, COPD assessment test; CI, confidence interval; COPD, chronic obstructive pulmonary disease; E-RS, evaluating respiratory symptoms-COPD; FP, fractional polynomial; LS, least squares; MMRM, mixed model repeated measures; SAC, self-administered computerised; TDI, transition dyspnoea index; UMEC, umeclidinium; VI, vilanterol.

**Figure 5.**
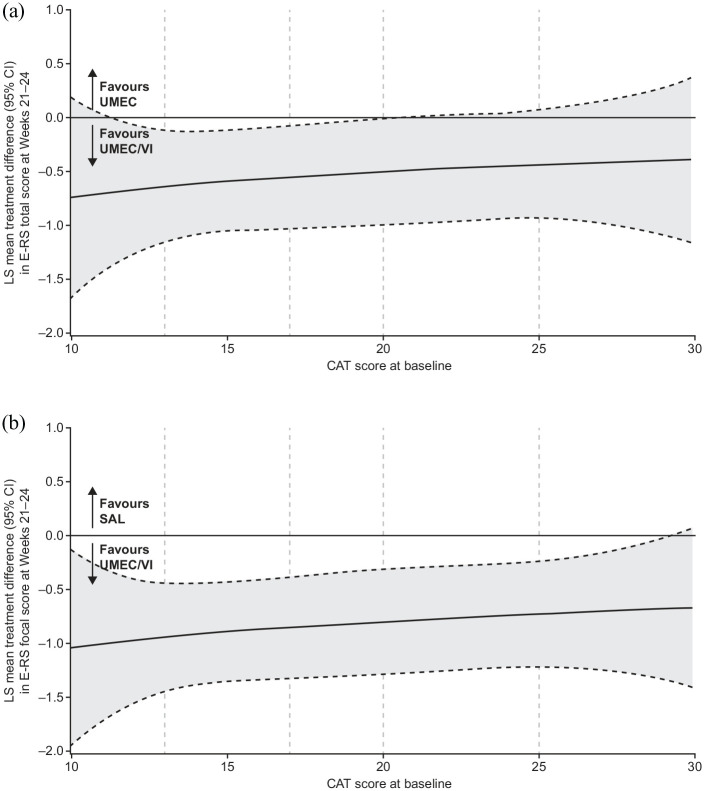
Improvement in 4-weekly E-RS total score at weeks 21–24 by baseline CAT score with UMEC/VI *versus* (a) UMEC and (b) SAL. Vertical dotted lines indicate quintiles of CAT score at baseline. FP analyses were conducted across baseline CAT scores of 10–30 as the study population included few patients with scores outside this range. The fitted MMRM included covariates of baseline E-RS score, geographical region, number of bronchodilators per day during run-in, 4-weekly period, treatment, FP1, FP2, and 4-weekly period by baseline E-RS score, 4-weekly period by treatment, FP1*treatment and FP2*treatment interactions. CAT, COPD assessment test; CI, confidence interval; COPD, chronic obstructive pulmonary disease; E-RS, evaluating respiratory symptoms-COPD; FP, fractional polynomial; LS, least squares; MMRM, mixed model repeated measures; TDI, transition dyspnoea index; SAL, salmeterol; UMEC, umeclidinium; VI, vilanterol.

Improvements in the SAC-TDI score at week 24 and E-RS total score were observed across the assessed CAT scores, with the greatest improvements seen with UMEC/VI (Supplemental Figure 2B, C). Greater numerical improvements in the SAC-TDI focal score at week 24 in favour of UMEC/VI *versus* UMEC and SAL were also observed across CAT scores. The 95% CI for these improvements excluded 0 across baseline CAT scores of approximately 12–21 *versus* UMEC and approximately 12–25 *versus* SAL (Figure 4). Similar patterns were observed for E-RS total score at weeks 21–24 (Figure 5).

#### Rescue medication use

**Figure 6.**
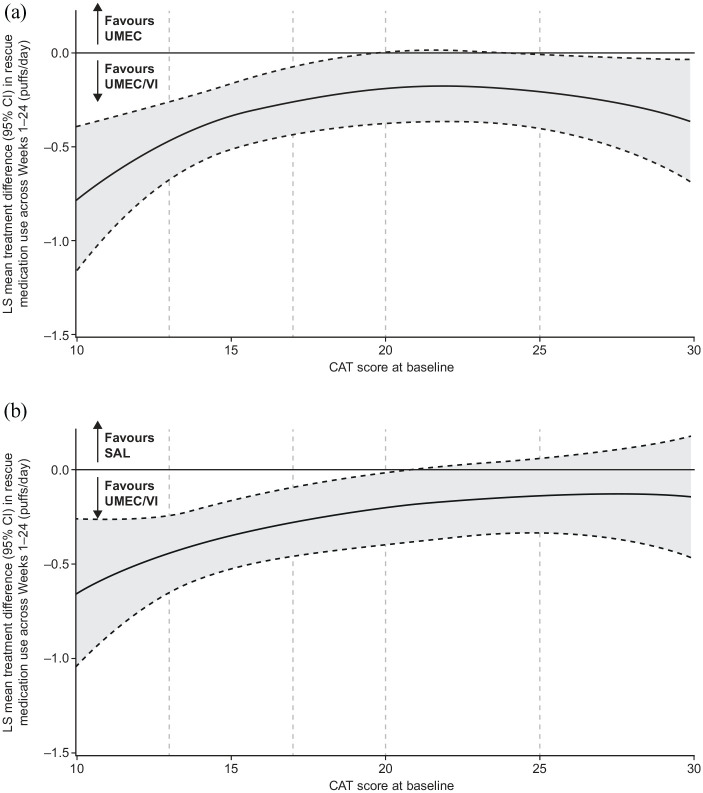
Improvement in rescue medication use (puffs/day) across weeks 1–24 by baseline CAT score with UMEC/VI *versus* (a) UMEC and (b) SAL. Vertical dotted lines indicate quintiles of CAT score at baseline. FP analyses were conducted across baseline CAT scores of 10–30 as the study population included few patients with scores outside this range. The fitted MMRM included covariates of baseline mean rescue medication use (puffs/day), geographical region, number of bronchodilators per day during run-in, 4-weekly period, treatment, FP1, FP2, and 4-weekly period by baseline mean rescue medication use (puffs/day), 4-weekly period by treatment, FP1*treatment and FP2*treatment interactions. CAT, COPD assessment test; CI, confidence interval; COPD, chronic obstructive pulmonary disease; FP, fractional polynomial; LS, least squares; MMRM, mixed model repeated measures; SAL, salmeterol; UMEC, umeclidinium; VI, vilanterol.

Improvements from baseline in rescue medication puffs/day over weeks 1–24 were observed with all treatments (Supplemental Figure 2D). Numerically greater improvements with UMEC/VI *versus* UMEC and SAL were observed across CAT scores, and the 95% CI excluded 0 at scores <20 in both comparisons (Figure 6).

#### SGRQ and CAT

Improvements from baseline in CAT and SGRQ scores were similar with UMEC/VI and UMEC or SAL across CAT scores (Supplemental Figures 3 and 4).

## Discussion

In this analysis of the EMAX trial, UMEC/VI demonstrated improvements from baseline *versus* both UMEC and SAL for most outcomes assessed in both CAT subgroups (<20 and ⩾20). Overall, patients with less severe symptoms (CAT <20) demonstrated larger improvements with UMEC/VI *versus* UMEC and SAL. Consistent incremental improvements in FEV_1_, E-RS scores and reductions in rescue medication use were also observed for all treatments in the CAT ⩾20 subgroup, with UMEC/VI demonstrating the largest improvements from baseline. UMEC/VI was associated with a consistent reduction in the risk of short-term deterioration compared with UMEC and SAL in both CAT subgroups. A complementary fractional polynomial analysis with the CAT score as a continuous variable, without imposing arbitrary cut-points, also revealed improvements in lung function and symptoms, as well as reductions in rescue medication use, favouring UMEC/VI *versus* UMEC or SAL. Consistent with the subgroup analyses, fractional polynomial analyses showed the greatest certainty of incremental treatment benefit with UMEC/VI *versus* UMEC or SAL for most outcomes in patients with CAT scores in the range of 10–21. Fractional polynomial models are a promising addition to traditional subgroup analyses, and further studies using this exploratory analysis may reveal non-linear relationships between disease severity and outcomes relevant to treatment decisions in individual patients.

These findings indicate that dual bronchodilator treatment with UMEC/VI leads to greater improvements in lung function and symptoms *versus* UMEC and SAL monotherapy irrespective of baseline CAT score, at least among the symptomatic patient population recruited to this trial (CAT score ⩾10 at screening). Consistent with this, the patient subgroup with less severe symptoms at baseline (CAT <20) generally had a higher incidence of short-term deterioration than those with more severe symptoms (CAT ⩾20), particularly in the monotherapy treatment arms. Dual bronchodilator treatment should therefore be considered as initial maintenance therapy for patients with COPD across a broader range of symptom severities than just those with CAT scores ⩾20. Our findings suggest that patients with CAT scores lower than 20 may also benefit from dual bronchodilator therapy.

Previous studies have also demonstrated consistent benefits of LAMA/LABA treatment *versus* monotherapy across a range of symptom severities. A *post hoc* analysis of the PINNACLE trials demonstrated consistent improvements in lung function and rescue medication use in patients receiving glycopyrrolate/formoterol fumarate (LAMA/LABA) compared with glycopyrrolate (LAMA) across a range of CAT scores.^16^ A *post hoc* analysis of the OTEMTO studies showed improvements in health status, symptoms and lung function in patients receiving tiotropium/olodaterol (LAMA/LABA) *versus* tiotropium (LAMA) across a range of symptom severities.^22^ The latter study did show greater improvements in health status among patients with more severe *versus* less severe breathlessness at baseline, but this disparity may be attributable to the use of mMRC to assess baseline severity, differences in study length, the proportion of patients receiving concurrent ICS (which may impact the incremental efficacy of add-on LABA in COPD),^23,24^ and/or differences in efficacy within the LAMA/LABA class.^24,25^ Taken together, the existing evidence and the findings of the present study contrast with the current GOLD treatment strategy report, which recommends LAMA/LABA as initial therapy only in highly symptomatic patients (CAT ⩾20) or in patients who have severe breathlessness.^13^

Several limitations should be considered in the interpretation and generalisability of this analysis. As the study recruited patients with CAT scores ⩾10 at screening and low exacerbation risk, we are not able to comment on the use of dual *versus* monotherapy in patients with less severe symptoms or a higher exacerbation risk. However, the findings of this study are relevant to the treatment needs of the large number of symptomatic patients with low exacerbation risk (GOLD B) without concurrent ICS. The study was powered to detect changes in FEV_1_ and SAC-TDI score in the ITT population, and was not powered to assess outcomes by subgroup or to detect changes in other outcomes. Therefore, unlike in the ITT population, trends in treatment differences in the smaller subgroup analyses did not always reach statistical significance. Furthermore, the analyses for the CAT <20 subgroup and fractional polynomial modelling were performed *post hoc*, and fractional polynomial analyses were only conducted over baseline CAT scores of 10–30 due to the small numbers of patients with baseline CAT scores outside this range. In addition, the study compared UMEC/VI with SAL rather than VI, which was not used in this post-registration trial because it is not licensed as a monotherapy in any country.

## Conclusion

In symptomatic patients at low risk of exacerbations who were not receiving ICS, dual bronchodilator therapy with UMEC/VI provides greater benefits than UMEC and SAL monotherapy irrespective of the baseline CAT score. Fractional polynomial modelling of the CAT score as a continuous variable represents a promising addition to traditional subgroup analyses and may reveal non-linear associations relevant to treatment decisions. The greatest treatment differences favouring dual therapy were observed in patients with a baseline CAT score of <20, although benefits were seen up to a CAT score of 30. This suggests that dual bronchodilation may be considered as appropriate initial therapy for patients with COPD across a broad range of symptom severities, not only those with severe symptoms (CAT ⩾20).

## Supplemental Material

Author_Response_1 – Supplemental material for Impact of baseline COPD symptom severity on the benefit from dual *versus* mono-bronchodilators: an analysis of the EMAX randomised controlled trial Click here for additional data file. Supplemental material, Author_Response_1 for Impact of baseline COPD symptom severity on the benefit from dual *versus* mono-bronchodilators: an analysis of the EMAX randomised controlled trial by Claus F. Vogelmeier, Edward M. Kerwin, Leif H. Bjermer, Lee Tombs, Paul W. Jones, Isabelle H. Boucot, Ian P. Naya, David A. Lipson, Chris Compton, Neil Barnes and François Maltais in Therapeutic Advances in Respiratory Disease

Reviewer_1_v.1 – Supplemental material for Impact of baseline COPD symptom severity on the benefit from dual *versus* mono-bronchodilators: an analysis of the EMAX randomised controlled trial Click here for additional data file. Supplemental material, Reviewer_1_v.1 for Impact of baseline COPD symptom severity on the benefit from dual *versus* mono-bronchodilators: an analysis of the EMAX randomised controlled trial by Claus F. Vogelmeier, Edward M. Kerwin, Leif H. Bjermer, Lee Tombs, Paul W. Jones, Isabelle H. Boucot, Ian P. Naya, David A. Lipson, Chris Compton, Neil Barnes and François Maltais in Therapeutic Advances in Respiratory Disease

Reviewer_2_v.1 – Supplemental material for Impact of baseline COPD symptom severity on the benefit from dual *versus* mono-bronchodilators: an analysis of the EMAX randomised controlled trial Click here for additional data file. Supplemental material, Reviewer_2_v.1 for Impact of baseline COPD symptom severity on the benefit from dual *versus* mono-bronchodilators: an analysis of the EMAX randomised controlled trial by Claus F. Vogelmeier, Edward M. Kerwin, Leif H. Bjermer, Lee Tombs, Paul W. Jones, Isabelle H. Boucot, Ian P. Naya, David A. Lipson, Chris Compton, Neil Barnes and François Maltais in Therapeutic Advances in Respiratory Disease

Supplementary_Figures_and_Table – Supplemental material for Impact of baseline COPD symptom severity on the benefit from dual *versus* mono-bronchodilators: an analysis of the EMAX randomised controlled trial Click here for additional data file. Supplemental material, Supplementary_Figures_and_Table for Impact of baseline COPD symptom severity on the benefit from dual *versus* mono-bronchodilators: an analysis of the EMAX randomised controlled trial by Claus F. Vogelmeier, Edward M. Kerwin, Leif H. Bjermer, Lee Tombs, Paul W. Jones, Isabelle H. Boucot, Ian P. Naya, David A. Lipson, Chris Compton, Neil Barnes and François Maltais in Therapeutic Advances in Respiratory Disease
