# Supplementary material for: Impact of baseline COPD symptom severity on the benefit from dual versus mono-bronchodilators: an analysis of the EMAX randomised controlled trial
Source: Ther Adv Respir Dis. 2020 Nov 9;14:1753466620968500. doi: 10.1177/1753466620968500 (PMC7659027; doi:10.1177/1753466620968500)
Supplement: Reviewer_1_v.1 – Supplemental material for Impact of baseline COPD symptom severity on the benefit from dual versus mono-bronchodilators: an analysis of the EMAX randomised controlled trial [file Reviewer_1_v.1.pdf]

Reviewer 1 v.1

Comments to the Author

This is a post hoc analysis of EMAX trial. The authors aimed to compare treatment responses between high and low symptom burden patients. Umeclidinium/vilanterol demonstrated favorable improvement compared with umeclidinium and salmeterol for the majority of outcomes irrespective of baseline CAT score. This study is very interesting and the result is novel. However, I have some comments

Major comments

1. Figure label is wrong. Figure 6 should be changed to figure 2. Figure 2 should be figure 3. Figure 3 should be figure 4. Figure 4 should be figure 5. Figure 5 should be figure 6.

2. Was there any statistical differences in baseline between patients with CAT<20 and CAT>=20?

3. I am wondering if the treatment response differed between patients with treatment naive and with already LABA or LAMA? About one third of patients were treatment naive during the run-in. Can the authors perform subgroup analysis for these patients?

4. In figure 6 (which should be figure 2), the incidence of CID in CAT<20 is higher than CAT>=20. For example, CID incidence in UV group with CAT<20 is 56%, 54%, and 66% (according to multiple definitions of CID). CID incidence in CAT>=20 is 54%, 48%, and 60%. Can the authors explain this result? Why more symptom group experienced less CID? Usually high CAT is associated with poor prognosis.
