# Supplementary material for: Impact of baseline COPD symptom severity on the benefit from dual versus mono-bronchodilators: an analysis of the EMAX randomised controlled trial
Source: Ther Adv Respir Dis. 2020 Nov 9;14:1753466620968500. doi: 10.1177/1753466620968500 (PMC7659027; doi:10.1177/1753466620968500)
Supplement: Reviewer_2_v.1 – Supplemental material for Impact of baseline COPD symptom severity on the benefit from dual versus mono-bronchodilators: an analysis of the EMAX randomised controlled trial [file Reviewer_2_v.1.pdf]

Reviewer 2 v.1

Comments to the Author

This is a study evaluated the benefits of dual BD vs monoTx in symptomatic COPD patients at low risk of exacerbation with a range of symptom severity who were not receiving ICS

The main findings were that consistent improvement in lung function, symptoms and reduction in rescue medication use with dual BD (UMEC/VI) vs UMEC or salmeterol (SAL) monoTx groups, with the largest benefits in patients with CAT scores 10-21.

After reading this article I have some questions and comments

Major comments:

1. In the table2, mean change from baseline for SAC-TDI and E-RS total score with UMEC/VI vs UMEC was not significantly different in both CAT<20 and CAT≥20 subgroups. The proportions of responders for SAC-TDI and CAT favored UMEC/VI vs UMEC only in the CAT <20 subgroup, not in the CAT≥20 subgroup. In addition, UMEC/VI did not showed significant better response compared with UMEC on the use of rescue medication and GADS in the CAT≥20 subgroup. Improvement from baseline in CAT and SGRQ were similar with UMEC/VI and UMEC or SAL. These results suggest that patients with higher degrees of baseline symptoms are not likely to have additional clinical benefit with dual BD compared with mono Tx group. It could be interpreted that UMEC/VI may have benefit only in patients with medium impact level of symptom burden. Please discuss this point.

2. Although authors concluded “Patients with symptomatic COPD benefit similarly from dual BD Tx with UMEC/VI, whether they have moderate or high symptom burden at baseline”, because of above reason, this has to be change to “Patients with symptomatic COPD benefit from dual BD Tx, with UMEC/VI, especially in patients with moderate symptom burden at baseline”

3. It would be more reasonable to conduct additional analyses to evaluate the robustness of the main finding using different baseline symptom burden cutoffs (ex mMRC scores 0-1 and ≥2, BDI cut-off scores of 0-4 and ≥5, E-RS score cut off?)

4. About 31% of study patients did not receive either LAMA or LABA during the 4-week run-in period, which involved the use of a placebo. Moreover, 39% did not receive maintenance Tx in the CAT≥20 subgroup during run-in. Although eligible patients were highly symptomatic, what was the reason that no maintenance medication was allowed during 4-week run-in in these patients? Please describe on this ethical point.

5. Please provide the data of previous COPD maintenance drug before study entry.

6. Please describe and summarize the clinical significance of measuring GADS score in COPD. Is it validated assessment tool in COPD? What the GADS is minimum clinically important difference (MCID) in COPD?
