# Supplementary material for: Impact of baseline COPD symptom severity on the benefit from dual versus mono-bronchodilators: an analysis of the EMAX randomised controlled trial
Source: Ther Adv Respir Dis. 2020 Nov 9;14:1753466620968500. doi: 10.1177/1753466620968500 (PMC7659027; doi:10.1177/1753466620968500)
Supplement: Supplementary_Figures_and_Table – Supplemental material for Impact of baseline COPD symptom severity on the benefit from dual versus mono-bronchodilators: an analysis of the EMAX randomised controlled trial [file Supplementary_Figures_and_Table.pdf]

Supplementary Figure S1 Proportion of responders for SGRQ and CAT with UMEC/VI versus **(a)** UMEC and **(b)** SAL

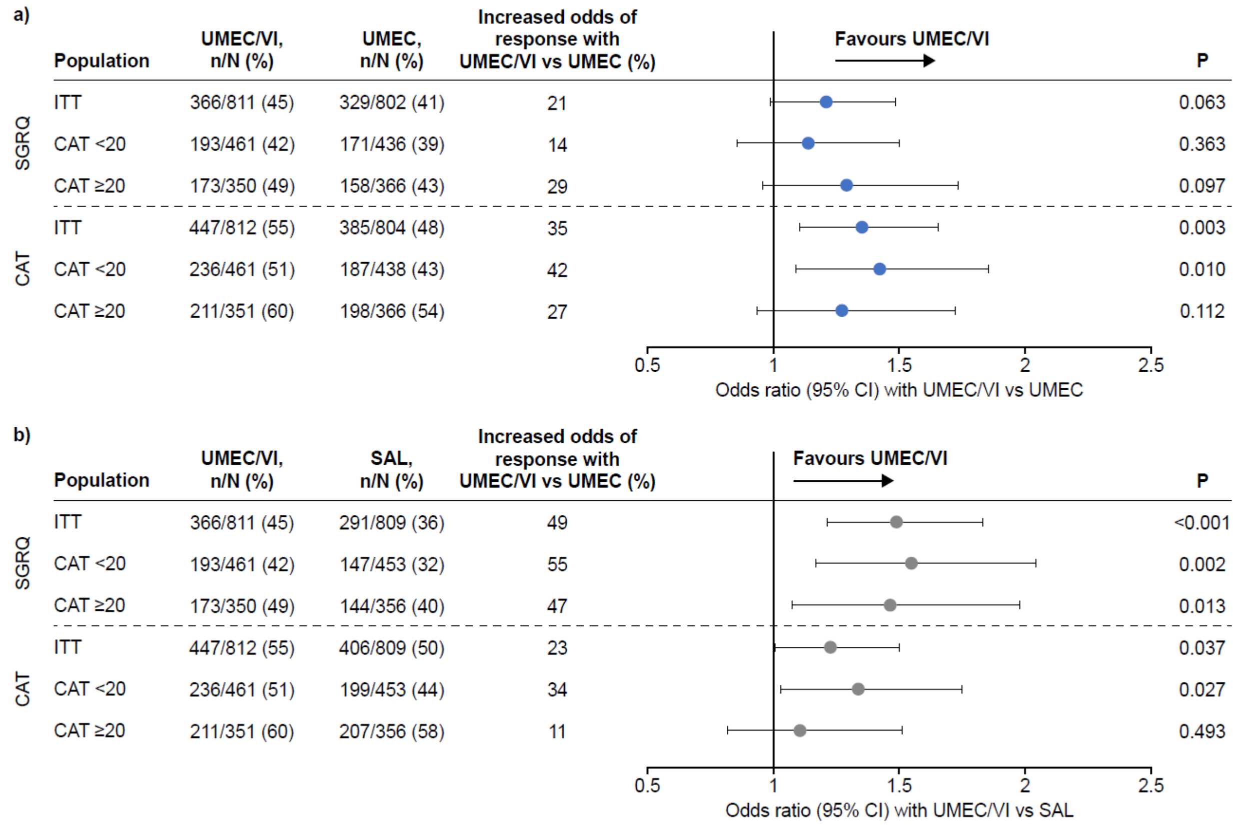

Analysis conducted using a generalised linear model with treatment as an explanatory variable and covariates of visit, baseline SGRQ score/baseline CAT score, number of bronchodilators received during run-in (0 or 1), geographical region, and visit by baseline SGRQ score/baseline CAT score and visit by treatment interactions.

CAT, COPD Assessment Test; CI, confidence interval; COPD, chronic obstructive pulmonary disease; ITT, intent-to-treat; SAL, salmeterol; SGRQ, St George's Respiratory Questionnaire; UMEC, umeclidinium; VI, vilanterol.

Supplementary Figure S2 Change from baseline with UMEC/VI, UMEC, and SAL by baseline CAT score in **(a)** trough FEV_1 at Week 24, **(b)** SAC-TDI focal score at Week 24, **(c)** 4-weekly E-RS total score at Weeks 21–24, and **(d)** rescue medication puffs/day over Weeks 1–24

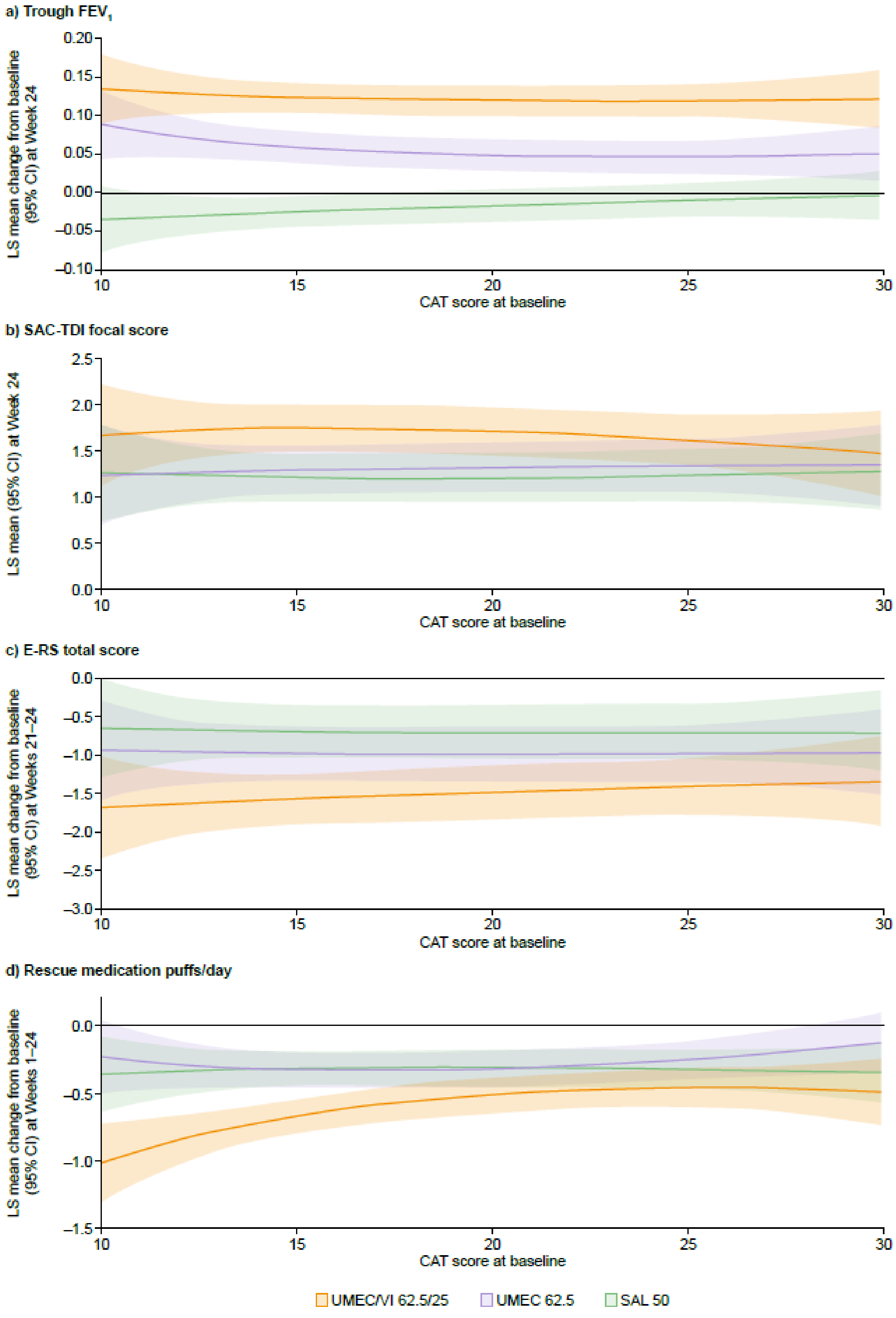

FP analyses were conducted across baseline CAT scores of 10–30 as the study population included few patients with scores outside this range. The fitted MMRM included covariates of (A, B) baseline

FEV_1/SAC-BDI, geographical region, number of bronchodilators per day during run-in, visit, treatment, FP1, FP2, and visit by baseline FEV_1/SAC-BDI, visit by treatment, FP1*treatment and FP2*treatment interactions; (C, D) baseline E-RS score/baseline mean rescue medication use (puffs/day), geographical region, number of bronchodilators per day during run-in, four-weekly period, treatment, FP1, FP2, and four-weekly period by baseline E-RS score/baseline mean rescue medication use (puffs/day), four-weekly period by treatment, FP1*treatment and FP2*treatment interactions.

BDI, Baseline Dyspnoea Index; CAT, COPD Assessment Test; CI, confidence interval; E-RS, Evaluating Respiratory Symptoms-COPD; FP, fractional polynomial; FEV_1, forced expiratory volume in 1 second; LS, least squares; SAC-TDI, self-administered computerised Transition Dyspnoea Index; SAL, salmeterol; UMEC, umeclidinium; VI, vilanterol.

Supplementary Figure S3 Improvement in SGRQ score at Week 24 by baseline CAT score with UMEC/VI versus **(a)** UMEC and **(b)** SAL

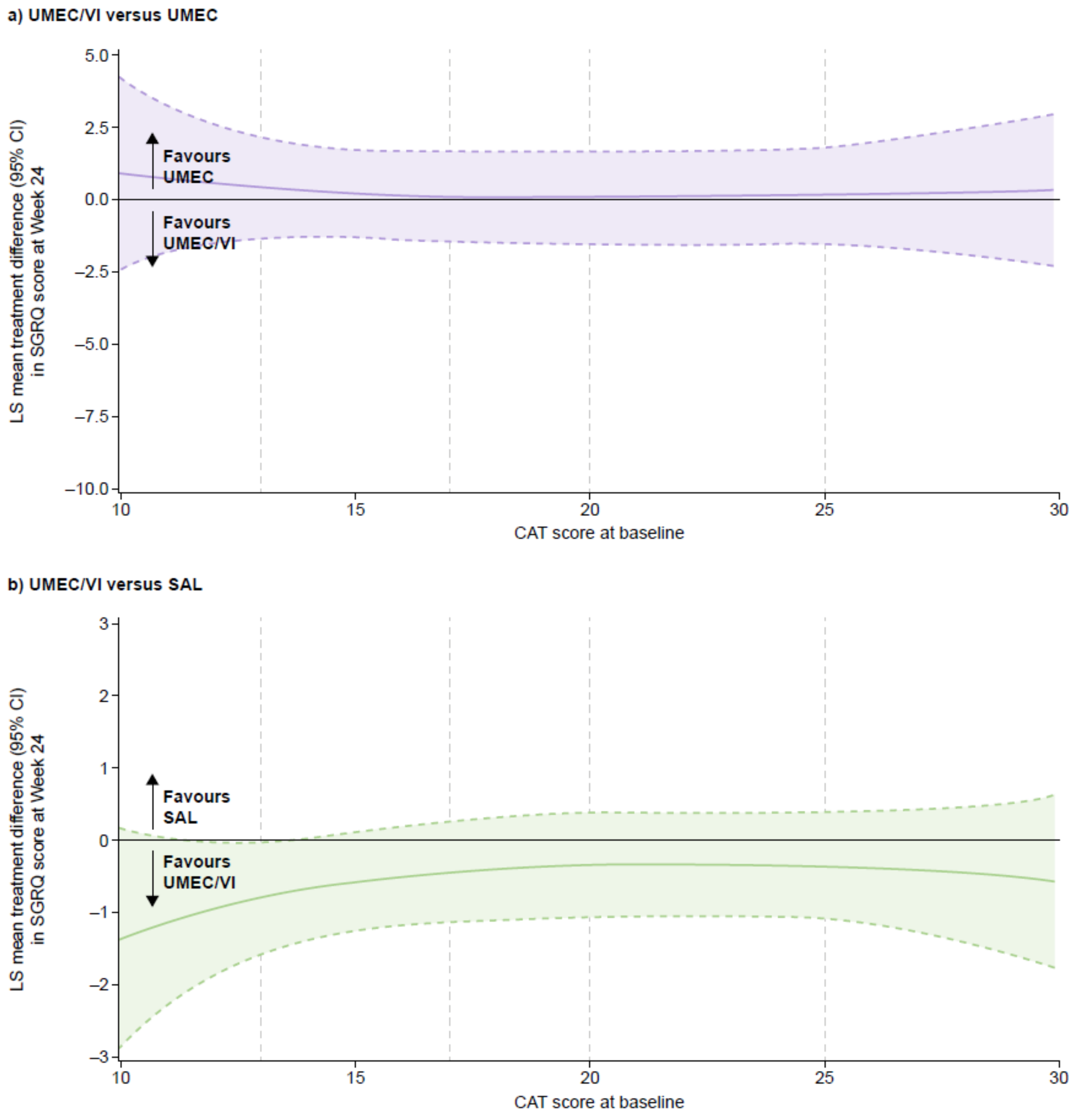

Vertical dotted lines indicate quintiles of CAT score at baseline. FP analyses were conducted across baseline CAT scores of 10–30 as the study population included few patients with scores outside this range. The fitted MMRM included covariates of baseline SGRQ score, geographical region, number of bronchodilators per day during run-in, visit, treatment, FP1, FP2, and visit by baseline SGRQ score, visit by treatment, FP1*treatment and FP2*treatment interactions.

CAT, COPD Assessment Test; CI, confidence interval; COPD, chronic obstructive pulmonary disease; FP, fractional polynomial; LS, least squares; SAL, salmeterol; SGRQ, St George's Respiratory Questionnaire; UMEC, umeclidinium; VI, vilanterol.

Supplementary Figure S4 Improvement in CAT score at Week 24 by baseline CAT score with UMEC/VI versus **(a)** UMEC and **(b)** SAL

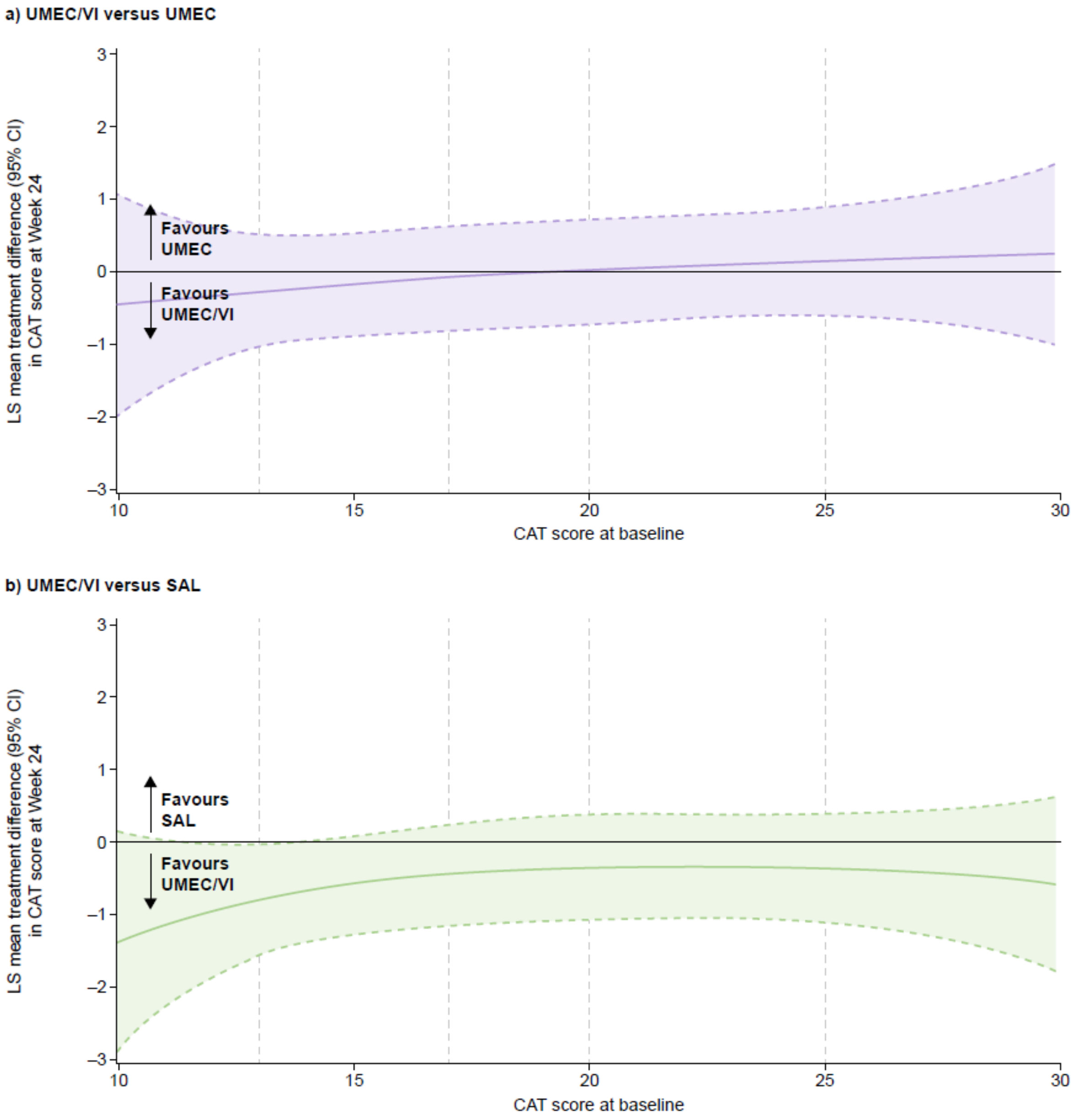

Vertical dotted lines indicate quintiles of CAT score at baseline. FP analyses were conducted across baseline CAT scores of 10–30 as the study population included few patients with scores outside this range. The fitted MMRM included covariates of baseline CAT score, geographical region, number of bronchodilators per day during run-in, visit, treatment, FP1, FP2, and visit by baseline CAT score, visit by treatment, FP1*treatment and FP2*treatment interactions.

CAT, COPD Assessment Test; CI, confidence interval; COPD, chronic obstructive pulmonary disease; FP, fractional polynomial; LS, least squares; SAL, salmeterol; UMEC, umeclidinium; VI, vilanterol.

Supplementary Table 1 CONSORT checklist

Section/Topic	Item No	Checklist item	Reported on page No
**Title and abstract**			
	1a	Identification as a randomised trial in the title	1
	1b	Structured summary of trial design, methods, results, and conclusions (for specific guidance see CONSORT for abstracts)	2
**Introduction**			
Background and objectives	2a	Scientific background and explanation of rationale	3
	2b	Specific objectives or hypotheses	4
**Methods**			
Trial design	3a	Description of trial design (such as parallel, factorial) including allocation ratio	4 and ref. 8 (primary manuscript)
	3b	Important changes to methods after trial commencement (such as eligibility criteria), with reasons	N/A
Participants	4a	Eligibility criteria for participants	4 and ref. 8 (primary manuscript)
	4b	Settings and locations where the data were collected	Ref. 8 (primary manuscript)
Interventions	5	The interventions for each group with sufficient details to allow replication, including how and when they were actually administered	4 and ref. 8 (primary manuscript)
Outcomes	6a	Completely defined pre-specified primary and secondary outcome measures, including how and when they were assessed	4–5 and ref. 8 (primary manuscript)
	6b	Any changes to trial outcomes after the trial commenced, with reasons	N/A
Sample size	7a	How sample size was determined	Ref. 8 (primary manuscript)
	7b	When applicable, explanation of any interim analyses and stopping guidelines	N/A

Randomisation:			
Sequence generation	8a	Method used to generate the random allocation sequence	Ref. 8 (primary manuscript)
	8b	Type of randomisation; details of any restriction (such as blocking and block size)	Ref. 8 (primary manuscript)
Allocation concealment mechanism	9	Mechanism used to implement the random allocation sequence (such as sequentially numbered containers), describing any steps taken to conceal the sequence until interventions were assigned	Ref. 8 (primary manuscript)
Implementation	10	Who generated the random allocation sequence, who enrolled participants, and who assigned participants to interventions	Ref. 8 (primary manuscript)
Blinding	11a	If done, who was blinded after assignment to interventions (for example, participants, care providers, those assessing outcomes) and how	Ref. 8 (primary manuscript)
	11b	If relevant, description of the similarity of interventions	N/A
Statistical methods	12a	Statistical methods used to compare groups for primary and secondary outcomes	5–6
	12b	Methods for additional analyses, such as subgroup analyses and adjusted analyses	5–6
**Results**			
Participant flow (a diagram is strongly recommended)	13a	For each group, the numbers of participants who were randomly assigned, received intended treatment, and were analysed for the primary outcome	Ref. 8 (primary manuscript)
	13b	For each group, losses and exclusions after randomisation, together with reasons	Ref. 8 (primary manuscript)
Recruitment	14a	Dates defining the periods of recruitment and follow-up	Ref. 8 (primary manuscript)
	14b	Why the trial ended or was stopped	N/A
Baseline data	15	A table showing baseline demographic and clinical characteristics for each group	Table 1

Numbers analysed	16	For each group, number of participants (denominator) included in each analysis and whether the analysis was by original assigned groups	6–11, all figures and tables
Outcomes and estimation	17a	For each primary and secondary outcome, results for each group, and the estimated effect size and its precision (such as 95% confidence interval)	6–11, all figures and tables
	17b	For binary outcomes, presentation of both absolute and relative effect sizes is recommended	6–11, all figures and tables
Ancillary analyses	18	Results of any other analyses performed, including subgroup analyses and adjusted analyses, distinguishing pre-specified from exploratory	6–11, all figures and tables
Harms	19	All important harms or unintended effects in each group (for specific guidance see CONSORT for harms)	Ref. 8 (primary manuscript)
**Discussion**			
Limitations	20	Trial limitations, addressing sources of potential bias, imprecision, and, if relevant, multiplicity of analyses	13
Generalisability	21	Generalisability (external validity, applicability) of the trial findings	11–13
Interpretation	22	Interpretation consistent with results, balancing benefits and harms, and considering other relevant evidence	11–13
**Other information**			
Registration	23	Registration number and name of trial registry	2
Protocol	24	Where the full trial protocol can be accessed, if available	N/A
Funding	25	Sources of funding and other support (such as supply of drugs), role of funders	14
