# Supplementary material for: Impact of baseline COPD symptom severity on the benefit from dual versus mono-bronchodilators: an analysis of the EMAX randomised controlled trial
Source: Ther Adv Respir Dis. 2020 Nov 9;14:1753466620968500. doi: 10.1177/1753466620968500 (PMC7659027; doi:10.1177/1753466620968500)
Supplement: Author_Response_1 – Supplemental material for Impact of baseline COPD symptom severity on the benefit from dual versus mono-bronchodilators: an analysis of the EMAX randomised controlled trial [file Author_Response_1.pdf]

**Comment**	**Author response**
**Reviewer 1**	
1. Figure label is wrong. Figure 6 should be changed to figure 2. Figure 2 should be figure 3. Figure 3 should be figure 4. Figure 4 should be figure 5. Figure 5 should be figure 6.	Apologies for this error, the figure numbering has been corrected.
2. Was there any statistical differences in baseline between patients with CAT=20?	Statistical comparisons of the baseline characteristics of patients in the CAT <20 and CAT ≥20 subgroups have been added to Table 1. These show that the CAT ≥20 subgroup had significantly worse lung function and higher symptom burden at baseline. In addition to the changes made to the table, the following text describing the baseline characteristics has been amended:<br>*"There were significant differences in the baseline characteristics of the CAT <20 and CAT ≥20 subgroups; for example, 24% and 39% of patients did not receive maintenance treatment during run-in, mean E-RS total scores were 8.4 and 13.4, and mean baseline SGRQ scores were 36.8 and 54.7, respectively (Table 1).* ***In addition,*** *~~the CAT <20 and CAT ≥20 subgroups at baseline~~, 593* ***(44%)*** *and 610* ***(57%) patients were current smokers*** *in the CAT <20 and CAT ≥20 subgroups at baseline, respectively. Overall, 317 (23%) and 308 (29%) patients were reversible to salbutamol in the CAT <20 and CAT ≥20 subgroups, respectively. Mean rescue medication use was 1.6 and 2.8 puffs/day in the CAT <20 and CAT ≥20 subgroups, respectively. ~~24% and 39% did not receive maintenance treatment during run-in, mean E-RS total score was 8.4 and 13.4, mean rescue medication use was 1.6 and 2.8 puffs/day, mean baseline SGRQ score was 36.8 and 54.7, and mean CAT score was 14.7 and 24.9 (Table 1).~~"*
3. I am wondering if the treatment response differed between patients with treatment naive and with already LABA or LAMA? About one third of patients were treatment naive during the run-in. Can the authors perform subgroup analysis for these patients?	We have performed subgroup analyses of patients who were receiving a LAMA or LABA in the 30 days prior to screening and those that were not. We presented some of these data at ATS 2019 (Bjermer L, et al. Am J Respir Crit Care Med. 2019;199:A3317). However, due to the large amount of data, these analyses are out of scope of this manuscript and we are currently developing a separate manuscript which presents these data.
4. In figure 6 (which should be figure 2), the incidence of CID in CAT=20. For example, CID incidence in UV group with CAT=20 is 54%, 48%, and 60%. Can the authors explain this result? Why more symptom group experienced less CID? Usually high CAT is associated with poor prognosis.	Thank you for your comment. As shown in Table 1, a greater proportion of patients in the CAT <20 versus the CAT ≥20 subgroup had a moderate exacerbation in the year prior to baseline (18% vs 14%; p=0.009). As exacerbations are a component of the CID endpoint, this may have contributed to the marginally higher CID rates seen in the CAT <20 versus the CAT ≥20 subgroup during the 24-week treatment period. However, it is important to note that the numerical differences between the incidence of CID in the CAT <20 and ≥20 subgroups are small (2−6%) and based on the confidence intervals we think that the data indicate that there are no differences in the risk of CID between the subgroups.

Reviewer 2	
1. In the table 2, mean change from baseline for SAC-TDI and E-RS total score with UMEC/VI vs UMEC was not significantly different in both CAT<20 and CAT≥20 subgroups. The proportions of responders for SAC-TDI and CAT favored UMEC/VI vs UMEC only in the CAT <20 subgroup, not in the CAT≥20 subgroup. In addition, UMEC/VI did not showed significant better response compared with UMEC on the use of rescue medication and GADS in the CAT≥20 subgroup. Improvement from baseline in CAT and SGRQ were similar with UMEC/VI and UMEC or SAL. These results suggest that patients with higher degrees of baseline symptoms are not likely to have additional clinical benefit with dual BD compared with mono Tx group. It could be interpreted that UMEC/VI may have benefit only in patients with medium impact level of symptom burden. Please discuss this point.	We agree that the symptomatic benefit of UMEC/VI versus UMEC appears to be smaller in patients with CAT scores ≥20 compared with <20, although both CAT subgroups did have significant improvement in lung function with UMEC/VI versus UMEC. However, it is important to note that analyses of the ITT population demonstrated statistically significant improvements in SAC-TDI, E-RS and GADS with UMEC/VI versus both UMEC and SAL (Maltais F et al. Resp Res 2019;20:238). Unlike the analyses of the ITT population (n=804−812 patients per treatment), the trial was not powered for subgroup analyses by baseline CAT score (CAT <20 subgroup n=438−461 patients per treatment; CAT ≥20 subgroup n=351−366 patients per treatment). The patient numbers have now been added to Table 2 to assist readers. The fractional polynomial analyses presented in Figures 4, 5 and 6 provide important additional information compared with the subgroup analyses that compare arbitrarily-defined subgroups. These indicate that the symptomatic benefits of UMEC/VI versus monotherapy are observed across the range of CAT scores but are generally greater in patients with lower CAT scores. It is difficult to speculate why this may be the case; however, it is important to consider that high CAT scores are associated with anxiety and depression (Harryanto H, et al. Int J COPD 2018;13:955–957; Miravitlles M, et al. Respir Med 2019;150:141–148), therefore, factors other than COPD symptoms may be driving the high CAT scores in some patients.
2. Although authors concluded "Patients with symptomatic COPD benefit similarly from dual BD Tx with UMEC/VI, whether they have moderate or high symptom burden at baseline", because of above reason, this has to be change to "Patients with symptomatic COPD benefit from dual BD Tx, with UMEC/VI, especially in patients with moderate symptom burden at baseline"	We think that caution is warranted before concluding that dual bronchodilation is less potent in highly symptomatic patients for a number of reasons: i) the study was not powered for this subgroup analysis; ii) for several outcome measures, although statistical significance was not reached (possibly due to the analyses being under-powered) the magnitude of improvement is similar between patients in both CAT <20 and ≥20 subgroups; iii) the fractional polynomial analyses suggest there are benefits of dual therapy across the range of CAT scores. Also, in the main conclusion we state that *"The greatest treatment differences favouring dual therapy were observed in patients with a baseline CAT score of <20, although benefits were seen up to a CAT score of 30."*<br><br>The sentence questioned by the reviewer is the conclusion of the abstract; therefore, we have modified the abstract: "*Patients with symptomatic COPD benefit similarly from dual bronchodilator treatment with umeclidinium/vilanterol~~, whether they have moderate or high symptom burden at baseline~~.* ***Fractional polynomial analyses demonstrated the greatest treatment differences favouring dual therapy in patients with a CAT score <20, although benefits were seen up to scores of 30. This suggests that dual bronchodilation may be considered as initial***

	***therapy for patients across a broad range of symptom severities, not only those with severe symptoms (CAT ≥20)**.“*
3. It would be more reasonable to conduct additional analyses to evaluate the robustness of the main finding using different baseline symptom burden cutoffs (ex mMRC scores 0-1 and ≥2, BDI cut-off scores of 0-4 and ≥5, E-RS score cut off?)	We agree that similar analyses according to other measures of symptom burden may be of interest (although mMRC was not measured in the EMAX trial); however, we think that the analyses by CAT score are more clinically relevant as the GOLD strategy provides treatment recommendations based on CAT score.
4. About 31% of study patients did not receive either LAMA or LABA during the 4-week run-in period, which involved the use of a placebo. Moreover, 39% did not receive maintenance Tx in the CAT≥20 subgroup during run-in. Although eligible patients were highly symptomatic, what was the reason that no maintenance medication was allowed during 4-week run-in in these patients? Please describe on this ethical point.	As noted by the reviewer, patients were permitted to use a LAMA or LABA during the 30-day run-in period; patients receiving other maintenance therapies were not eligible for the study. It was the treating physicians' decision not to prescribe maintenance therapy in the 31% of patients who were not receiving a LAMA or LABA during the run-in period; no treatment was withheld from patients.
5. Please provide the data of previous COPD maintenance drug before study entry.	These data have been added to the Results:<br>***“During the run-in period, 1576/2425 (65%) of patients in the ITT population were receiving long-acting bronchodilator maintenance medication (LAMA: 1194/2425 [49%]; LABA: 404/2425 [17%]).“***
6. Please describe and summarize the clinical significance of measuring GADS score in COPD. Is it validated assessment tool in COPD? What the GADS is minimum clinically important difference (MCID) in COPD?	The GADS is not a validated assessment tool in COPD and does not have a minimum clinically important difference. Patients were asked to rate how their COPD had changed from baseline using a 7-point Likert scale of much better, better, slightly better, no change, slightly worse, worse and much worse. The GADS assessment was included in the EMAX trial as a simple tool to measure patient-perceived change in overall COPD severity, to complement the more detailed assessments provided by the more established patient-reported outcomes used in the trial.
